# Accurate segmentation of green fruit based on optimized mask RCNN application in complex orchard

**DOI:** 10.3389/fpls.2022.955256

**Published:** 2022-08-10

**Authors:** Weikuan Jia, Jinmeng Wei, Qi Zhang, Ningning Pan, Yi Niu, Xiang Yin, Yanhui Ding, Xinting Ge

**Affiliations:** ^1^School of Information Science and Engineering, Shandong Normal University, Jinan, China; ^2^Key Laboratory of Intelligent Control and Manufacturing of Agricultural Machinery, Wuyi University, Wuyishan, China; ^3^School of Informatics, University of Leicester, Leicester, United Kingdom; ^4^School of Agricultural Engineering and Food Science, Shandong University of Technology, Zibo, China; ^5^School of Medical Imaging, Xuzhou Medical University, Xuzhou, China

**Keywords:** mask RCNN, instance segmentation, MobileNetv3, boundary patch refinement, green fruit

## Abstract

Fruit and vegetable picking robots are affected by the complex orchard environment, resulting in poor recognition and segmentation of target fruits by the vision system. The orchard environment is complex and changeable. For example, the change of light intensity will lead to the unclear surface characteristics of the target fruit; the target fruits are easy to overlap with each other and blocked by branches and leaves, which makes the shape of the fruits incomplete and difficult to accurately identify and segment one by one. Aiming at various difficulties in complex orchard environment, a two-stage instance segmentation method based on the optimized mask region convolutional neural network (mask RCNN) was proposed. The new model proposed to apply the lightweight backbone network MobileNetv3, which not only speeds up the model but also greatly improves the accuracy of the model and meets the storage resource requirements of the mobile robot. To further improve the segmentation quality of the model, the boundary patch refinement (BPR) post-processing module is added to the new model to optimize the rough mask boundaries of the model output to reduce the error pixels. The new model has a high-precision recognition rate and an efficient segmentation strategy, which improves the robustness and stability of the model. This study validates the effect of the new model using the persimmon dataset. The optimized mask RCNN achieved mean average precision (mAP) and mean average recall (mAR) of 76.3 and 81.1%, respectively, which are 3.1 and 3.7% improvement over the baseline mask RCNN, respectively. The new model is experimentally proven to bring higher accuracy and segmentation quality and can be widely deployed in smart agriculture.

## Introduction

Around the world, the planting area of orchards is very vast, and people all over the world have a large demand for fruit production. However, there are many production links of fruit, and the work content is huge and redundant. As the key of production link, fruit picking consumes a lot of human resources. To liberate the labor force and further expand the production scale of orchards, it is necessary to develop automatic mechanical production and management. Fruit picking is the most time-consuming and laborious link in the production process. The development of intelligent and automatic picking has become a necessary trend in the fruit production process (Luo et al., 2016; Bargoti and Underwood, 2017; Lin et al., 2021b; Zhang et al., 2021). As the key of automatic picking, visual recognition system can determine the efficiency of machine picking. The vision system aiming at accurate location and segmentation has important research significance for various automatic agricultural applications (Jana et al., 2017; Fu et al., 2020; Tang et al., 2020; Chen et al., 2021; Wu et al., 2022). Compared with manual picking, automatic machine picking can improve picking efficiency and save labor cost. However, the fruit growth environment is often complex. The recognition and location of target fruit by picking robot will be affected by the factors such as branch and leaf occlusion, dim light, cloudy and rainy weather, and equipment viewfinder angle, which limits the development of automatic picking operation. The development of an excellent vision system can enable the picking robot to obtain high-precision target fruit positioning and segmentation results in a complex orchard environment, reduce the phenomenon of false detection and missed detection caused by occlusion and the same color system with the background leaves, and reduce the damage to the fruit surface. Therefore, the instance segmentation model with strong robustness, stability, and high accuracy can further improve the picking efficiency, reduce the labor cost, and improve the yield of fruits and other agricultural products.

As the core field of artificial intelligence (Nyarko et al., 2018; Peng et al., 2018; Liu Q. et al., 2019; Jia et al., 2020), traditional machine learning has milestone significance for the development of image recognition. Machine learning methods first need to extract relevant features from many datasets, then learn the data through algorithms, and finally make prediction and decision, which is the key to the realization of artificial intelligence. Tian proposed a recognition algorithm based on the depth image. The gradient information is obtained from the depth image, and the segmentation algorithm is introduced into the red–green–blue (RGB) image. Finally, the center and maximum radius of the target fruit was scanned to fit the contour size of the target fruit, so as to realize apple recognition and location (Tian et al., 2019). Wu proposed a fruit point cloud segmentation algorithm integrating color and three-dimensional geometric features. First, the local descriptor is used to obtain the candidate fruit region, and then, the global descriptor is used to remove the background region. This method improves the fruit recognition ability of the picking robot, but it is difficult to obtain effective three-dimensional geometric features for irregular curved fruits, which makes the generalization ability of the algorithm weak (Wu et al., 2020). Saedi proposed a new model composed of multiple convolutional, max-pooling, global average pooling, and fully connected (Fc) layer to solve the problems of occlusion and light change in orchard environment. Through the optimization test, Nadam with the best effect is selected as the optimizer, which improves the accuracy and reduces the training parameters (Saedi and Khosravi, 2020). Aiming at the problem that the color of green fruit is similar to that of leaf, Sun proposed to roughly determine the fruit region using the attention-based information maximization algorithm and cut the identified apple region using the adaptive pixel expansion method to remove the background information, so as to realize the accurate segmentation of fruit target. However, for the connected highlight regions in the background, the segmentation effect is still not ideal (Sun et al., 2020). Feng proposed a forward-looking infrared camera based on multi-spectral dynamic imaging technology to obtain fruit images. The candidate fruit region is located based on the pseudocolor and texture information of multi-spectral dynamic image (Feng et al., 2019). Most of the fruit recognition methods based on machine learning recognize and segment the fruit based on the texture features, color, boundary shape, grayscale value, and so on. However, the real orchard environment is often complex, and the picking robot will encounter various difficulties in the complex orchard environment. For example, the same color system between background leaves and green fruits makes it difficult to distinguish colors, the serious occlusion and overlap between fruits lead to incomplete fruit shape, and the change of light intensity leads to unclear fruit representation. These problems seriously hinder the development of machine learning.

With the increasing volume and complexity of application data, the method of manually extracting image features from data by machine learning cannot deal with the problem quickly and simply. As a new branch derived from machine learning, deep learning does not need to extract features manually, and there are many layers of neural network. In theory, it can be mapped to any function to solve more complex problems (Hussain et al., 2020; Naranjo-Torres et al., 2020; Jia et al., 2021; Lin et al., 2021a). Ilyas proposed convolutional encoder–decoder network for strawberry fruit maturity recognition and diseased fruit and introduced adaptive receptive field, channel selection module, and bottleneck module to realize the accurate recognition of strawberry fruit, but the model could not segment a single target (Ilyas et al., 2021). Aiming at many problems in complex orchard environment, Kang proposed a one-stage detector DaSNet-v2. DaSNet-v2 used the lightweight backbone network (LW-Net) to realize the tasks of fruit detection and instance segmentation, and semantic segmentation of branches. The three recognition tasks are combined into a single network architecture to realize the accurate recognition of fruits (Kang and Chen, 2020). To predict crop pests and diseases at an early stage, Kavitha Lakshmi et al. proposed an improved framework for detecting plant diseases based on pixel-level mask region convolutional neural networks. The model can effectively locate and segment the pest and disease sites of crops by adjusting the proportion of anchor frames and backbone structure in the region generation network to improve the accuracy and speed (Kavitha and Savarimuthu, 2021). To detect the morphological features of leaves and plant specimens, Triki proposed an instance segmentation method based on deep learning, namely, deep-leaf. This method is used to improve the leaf detection and pixel segmentation based on the traditional mask RCNN model. It can segment the leaves of different families, measure the length and width of the leaves, and reduce the recognition error. However, the blade detection with missing shape is not considered (Triki et al., 2021). The recognition accuracy of the instance segmentation model based on deep learning is significantly improved compared with that based on machine learning. Specifically for algorithms with a large amount of data, deep learning can solve more complex problems with a simple end-to-end processing method. However, the above methods based on deep learning are still not accurate enough for the recognition and segmentation effect of target fruit. The limitations and benefits of previous studies are listed in Table 1.

**Table 1:** Overview of previous research work.

**Algorithm**	**Algorithm overview**	**Variants for fruit recognition**	**Benefits**	**Limitations**
Support Vector Machine (SVM)	SVM is a linear binary classifier with the largest interval defined in feature space	Fruit detection and grading algorithm (Bhargava and Bansal, 2020); SVM classifier algorithm (Patel et al., 2019)	A. Strong robustness; B. High accuracy	A. Unable to multi classify; B. High memory need
Cluster Analysis (CA)	CA is the analytical process of grouping datasets into multiple classes consisting of similar objects	K-means clustering algorithm (Pham and Lee, 2015; Sidehabi et al., 2018); Density peaks clustering algorithm (Wang et al., 2017; Guan et al., 2021)	A. Fast convergence speed of the algorithm; B. Easy and efficient algorithm	A. Poor anti-interference ability; B. Clustering centers need artificial intervention and can easily lead to subjective errors
Convolutional Neural Network (CNN)	CNN is a deep neural network with convolutional structure, which can be directly used for image processing	Deep leaf algorithm (Triki et al., 2021); Based mask RCNN (Liu X. et al., 2019; Yu et al., 2019); FoveaMask (Jia et al., 2021); Dense to detection algorithm (Wei et al., 2022)	A. Anchor-based methods have high accuracy; B. Anchor-free methods have low complexity and faster speed	A. Unable to balance speed and accuracy; B. Poor recognition of small and occluded objects
Generative Adversarial Network (GAN)	Generating networks and discriminating networks interact to learn the distribution of data	GAN data augmentation (Bird et al., 2022); GAN review (Olaniyi et al., 2022)	A. Fast sample generation; B. Reduce data preparation	A. Model training instability; B. The model is not easy to converge

To improve the segmentation quality and recognition accuracy of the model, the flexible MobileNetv3 (Howard et al., 2019) is proposed to be used as the backbone network of mask RCNN (He et al., 2017) to reduce the complexity of the algorithm, and the post-processing module boundary patch refinement (Tang et al., 2021) (BPR) is added at the end of the optimized mask RCNN model to improve the quality of the segmentation mask. The main contributions of the new model are as follows:

1) A lightweight backbone network, MobileNetv3, is used in the new model, which is able to reduce the computational complexity of the two-stage model, reduce the storage resource requirements of the picking robot, and improve the accuracy of the algorithm.
2) A plug-and-play BPR post-processing module is added to the new model to optimize the generated coarse masks, which can effectively correct the erroneous pixels and obtain higher resolution feature maps to improve accuracy.
3) In this study, persimmon dataset was produced to validate the accuracy results of the model, and ablation experiments were conducted with other segmentation models.
4) The new model has strong generalization ability and can be effectively deployed to other automatic fruit picking scenarios, such as apples, cucumbers, and other crops.

The subsequent sections of this paper are organized as follows: In Section Green fruit datasets, the process of making the perspex dataset is described in detail, and some of the data images are shown and introduced. The general structure of the new model is presented in a general divisional structure in Section Overall structure of model. Following the flow of the model, first, the lightweight backbone network MobileNetv3 of the new model is introduced in Section Backbone network MobileNetv3. The prediction output process of the model is presented in Section Mask output based on the optimized Mask RCNN. The process of optimizing the coarse mask of the instance boundary by the BPR module is described in Section Boundary patch refinement post-processing module. The loss function of the model is introduced in Section Loss function. In Section Data analysis and model comparison, the validation results of the new model and the visualization images are presented, and a comparison test with some other mainstream models is performed. A summary of this study is presented in Section Conclusion.

## Green fruit datasets

### Datasets collection and labeling

In this study, persimmons in the immature period were selected as the research object. The fruits in this period were green and round, which met the research requirements. The reason why green persimmon is selected as the research object in this study is to facilitate the subsequent full-time detection of fruits and lay a foundation for identifying other fruit varieties with similar growth environment.

Image collection object: Green persimmons in growing period, the persimmon varieties include “niuxin” persimmon, “jixinhuang” persimmon, “jingmian” persimmon, etc.

Image collection location: The mountain behind Shandong Normal University (Changqing campus) and the southern mountainous area of Jinan City.

Image collection equipment: Canon EOS 80D Single Lens Reflex camera. The camera used complex metal oxide semiconductor image sensor. The image resolution was 6,000 × 4,000 pixels, saved in JPG format, 24-bit color images.

Image collection environment: The single lens reflex camera was used to collect green fruit images from a variety of different angles in a real complex orchard environment. The shooting angles of the target fruit include front, side, close range, long-range, and other shooting angles. The image acquisition time was selected in the morning, noon, and night for three centralized acquisition in different time periods. In the early morning, fruit images were collected from various angles under soft light, and frost and dew may appear on the fruit. At noon, fruit images were collected at various shooting angles (including forward light, reverse light, and side light) in strong light environment. At night, fruit images were collected from various shooting angles under the environment of light emitting diode artificial auxiliary light source. The fruit images collected in three different time periods need to fully consider the complexity of orchard environment, ensure that the collected data are random and representative, and can maximize the real-time operation requirements of agricultural equipment.

**Figure 1:**
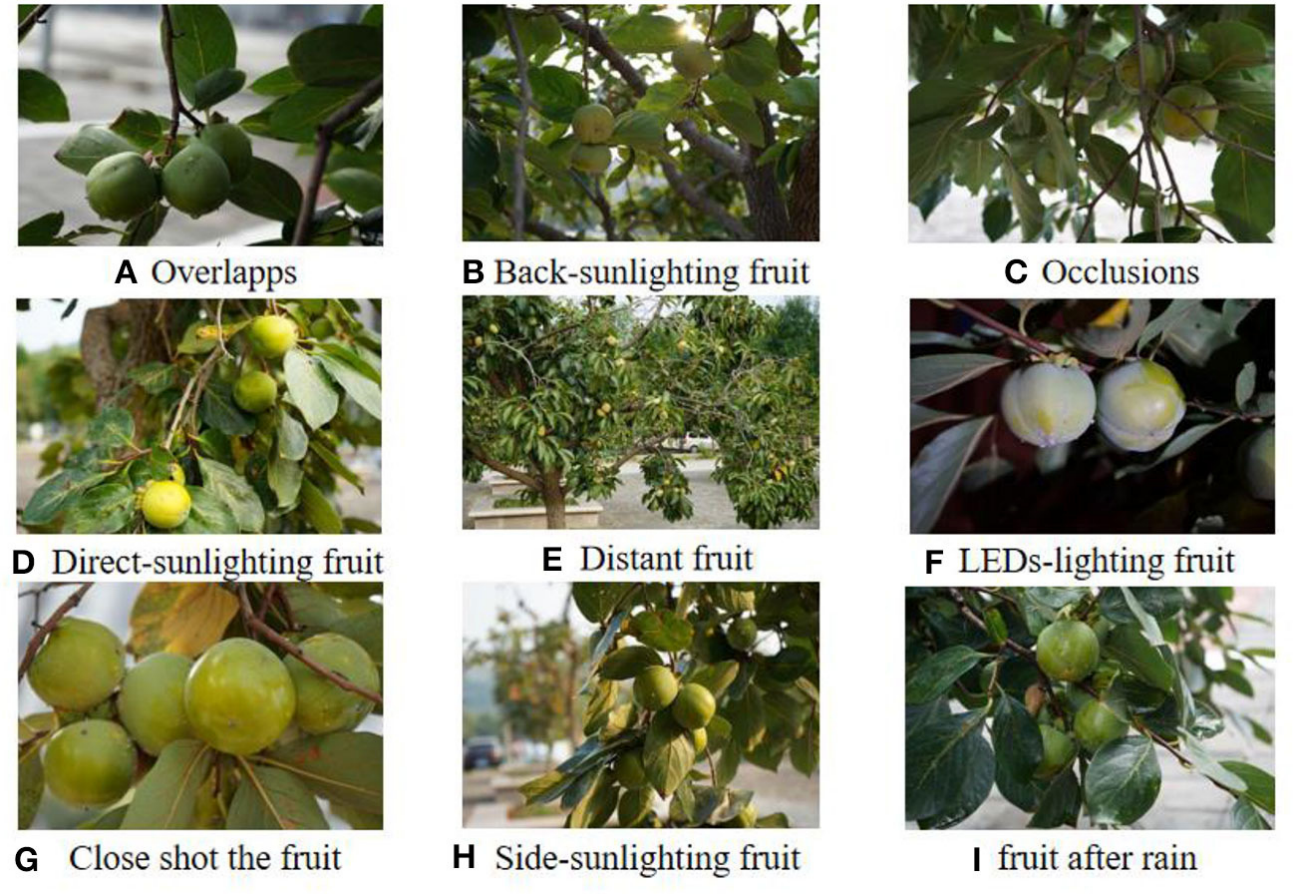
Green persimmon fruit images under different complex orchard environments. **(A)** Overlapps, **(B)** Back-sunlighting fruit, **(C)** Occlusions, **(D)** Direct-sunlighting fruit, **(E)** Distant fruit, **(F)** LEDs-lighting fruit, **(G)** Close shot the fruit, **(H)** Side- Back-sunlighting fruit, **(I)** Fruit after rain.

A total of 568 persimmon images were collected and preprocessed in this research experiment. First, gray processing was performed on the color image to reduce the amount of data to be processed. In this study, the mean grayscale method was adopted, and the RGB channel pixels were averaged as the grayscale value. The acquired data were then subjected to image enhancement to enhance the useful information of the image, improve the visual effect of the image, expand the differences between different object features in the image, and suppress the uninteresting features. In this study, the histogram equalization image enhancement method was used to nonlinearly stretch the image and redistribute the image pixel values, so that the number of pixels in a certain gray range is roughly the same. Finally, the contour and type of the target was labeled with LabelMe software, and the processed image was made into persimmon dataset. As shown in Figure 1, the dataset includes many complex situations, such as overlap, rainy day, backlight, occlusion, smoothing, side light, long-range, close-up, night view, and so on. The use of datasets containing many different complex environment images can make the research obtain more representative and convincing results. To meet the requirements of real-time orchard segmentation and reduce the follow-up experimental time, the image resolution was 6,000 × 4,000 pixels compressed to 600 × 400 pixels.

## Overall structure of model

To solve the problem that it is difficult for intelligent picking robot to accurately locate the target fruit and segment the target fruit with high quality in complex orchard environment, a two-stage instance segmentation method based on anchor frame is proposed in this study. The powerful backbone network and efficient rough mask post-processing module of the new model are beneficial to greatly improve the accuracy and robustness of the algorithm, so as to make the target fruit meet the requirements of high-quality and accurate segmentation.

**Figure 2:**
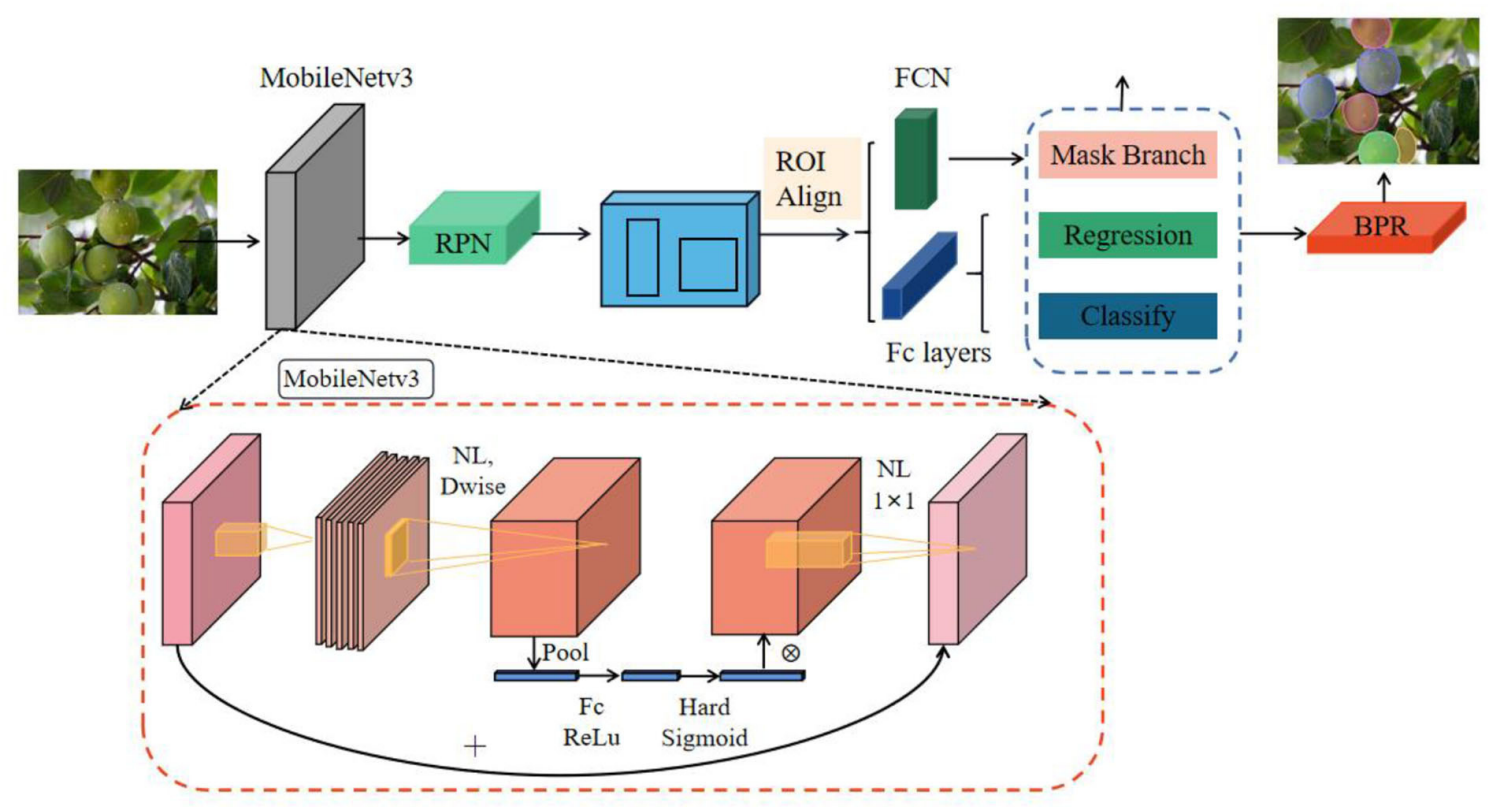
The overall structure of the optimized mask RCNN. The features of the input image are extracted through MobileNetv3 and FPN structure. The extracted features are used for subsequent classification, regression, and mask operations. Finally, the final segmentation result is obtained by optimizing the rough mask boundary of the boundary through the BPR module.

The classical mask RCNN, as an anchor two-stage segmentation model, has high computational complexity, large number of parameters, and slow speed, there is still much room for improvement in accuracy and efficiency, and the segmented target mask profile is relatively coarse. As shown in Figure 2, to solve these tricky problems, the new model uses MobileNetv3 as the backbone network to reduce the complexity of the model and further improve the accuracy of the model in identifying the target fruit. After the backbone network, the feature pyramid network (Lin et al., 2017) (FPN) is connected to realize multi-scale feature fusion. The detail information of the bottom layer and the semantic information of the top layer are fused to increase the low-level receptive field, so that the small target fruit can obtain more context information. The backbone structure of MobileNetv3 and FPN can be flexibly integrated into different segmentation models and bring more efficient and simple segmentation results. The optimized mask RCNN is used as a two-stage segmentation model. After feature extraction, region proposal network (RPN) is used to generate candidate region in the first stage. Additionally, in the second stage, the classification and regression of region of interest (ROI) are realized through the Fc layer. At the same time, the mask prediction branch of ROI is added in parallel with the classification branch and regression branch. The classification branch and regression branch use the Fc layer as the classifier, and the mask branch uses the Fc network to realize the binary mask segmentation of ROI. The segmentation mask generated by the optimized mask RCNN model is still rough, especially the segmentation of contour boundary is not smooth enough. For the small details of the instance edge, the processing is not accurate, and the segmented contour is not appropriate to the shape of the real target. Therefore, BPR post-processing module is used to optimize the rough mask boundary patches to generate more accurate and smooth contour information. Compared with the traditional mask RCNN model, the optimized model further improves the pixel resolution and mask segmentation quality.

### Backbone network MobileNetv3

**Figure 3:**
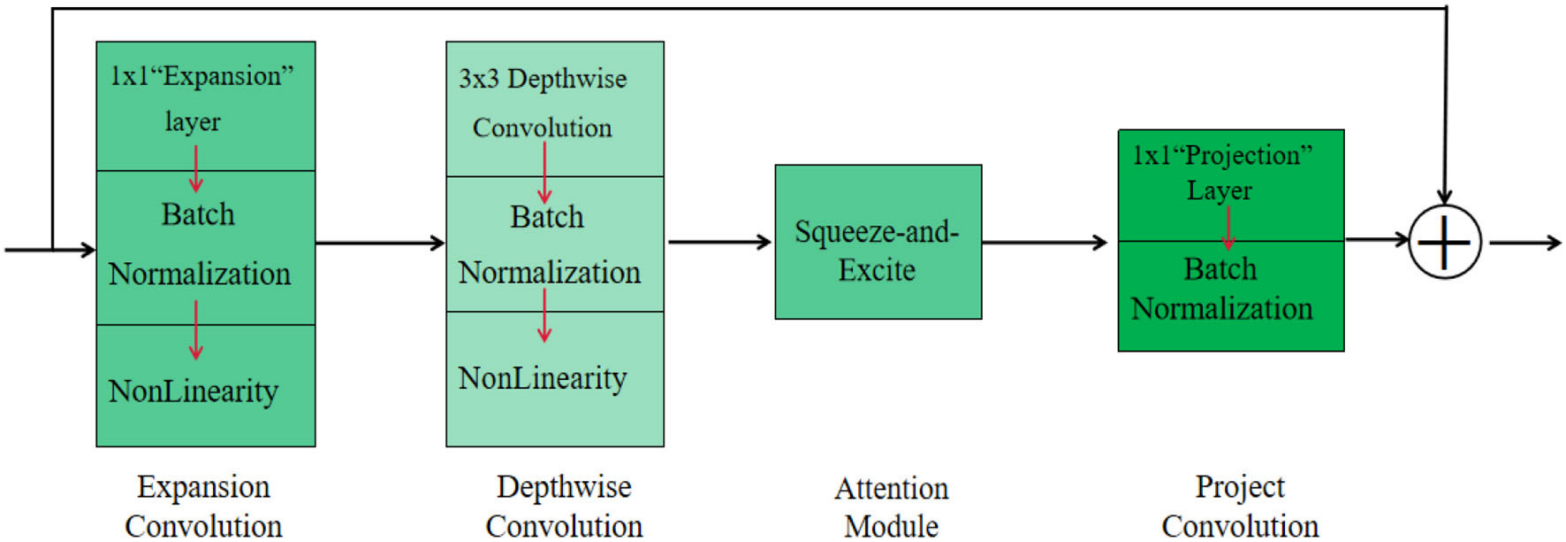
Overall structure of MobileNetv3 network.

A successful backbone network plays a crucial role in image processing, and a better backbone network should reduce the computational complexity while improving the accuracy and stability of the model. To meet the requirements of automated picking systems that can pick target fruits in real time and the storage resources required by mobile devices, the backbone network for vision systems should be a lightweight MobileNetv3 network. The MobileNetv3 network is a model framework that can be used on the embedded devices with a low number of parameters and meets the accuracy requirements compared to other lightweight networks. The model can be easily applied to segmentation or detection tasks. The overall structure of the Mobilenetv3 network is illustrated in Figure 3. The overall structure of this network differs from the residual network that first descends dimensionality and then raises dimensionality, but first raises dimensionality and then lowers dimensionality.

As shown in Figure 3, the overall structure of MobileNetv3 can be divided into four parts. In the first part, MobileNetv3 uses a 1 × 1 extension layer to extend the low dimension to the high dimension. It also uses batch normalization after the extension layer to avoid the gradient dispersion problem in the subsequent activation functions. Note that the nonlinearity here contains both hard-swish and ReLU activation functions, because the activation functions used are not consistent, so nonlinearity is used uniformly. The second part is a 3 × 3 depthwise convolution, also using batch normalization and nonlinearity after the structure. The third part is a squeeze-and-excite (Hu et al., 2018) module similar to the channel attention mechanism. Squeeze-and-excite, as a plug-and-play module, is able to increase the weights of useful channels to enhance the model's ability to extract features. The fourth part is the projection convolution, which uses a 1 × 1 projection layer and BN.

#### Squeeze-and-excitation

**Figure 4:**
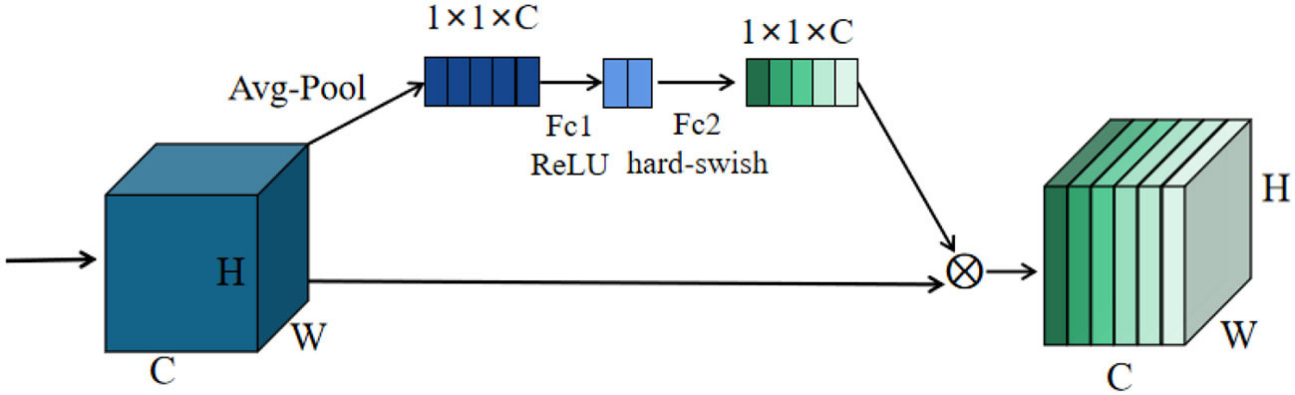
Overall structure of squeeze-and-excite.

Since each channel has different degrees of importance, an attention mechanism is introduced in this study to locate to the information of interest and suppress the unimportant information. The overall structure of the squeeze-and-excitation module is shown in Figure 4. First, the feature map is subjected to a global averaging pooling operation, which turns each two-dimensional feature map into a real number. It is equivalent to performing a squeeze operation to squeeze the size of the feature map from H×W×C to 1 × 1×C size. The feature map is then passed through Fc1, and the number of Fc1 nodes is 1/4 of the number of channels. ReLU is used as the activation function after Fc1. The number of nodes in Fc2 is the same as the previous number of channels. Finally, the real numbers of each channel are multiplied as weights with the corresponding feature map at the beginning.

#### Hard-swish activation function

In the previous Mobilenetv2 structure, ReLU6 was used as the activation function after the bottleneck structure. This study is inspired by swish (Elfwing et al., 2018), which can better improve the accuracy of the model. However, swish has the problem that it is complicated to calculate and derive, and it is not friendly to the quantization process. To solve this problem, the sigmoid function σ(*x*) in swish function is replaced by segmented linear hard analog. Therefore, in this paper, the hard-swish function is defined as the Equation (1):


(1) $$\begin{array}{r} Hard-swish\left[i\right]=i\frac{ReLU6\left(i+3\right)}{6} \end{array}$$

where ReLU = min[max(i,0),6].

#### Network layer details for MobileNetv3

To further understand the network structure of MobileNetv3, Table 2 lists the details of each network layer. In Table 2, the input feature size, the convolutional operation, the expanded dimension, and the output channel size for each layer of the network are shown in turn. In addition, the table enables to visualize whether the network uses the squeeze-and-excitation module and whether the nonlinearity used is hard-swish or ReLU. In addition, the stride of each network is given.

**Table 2:** Specification for MobileNetv3.

**Input**	**Operator**	**Exp size**	**Out**	**SE**	**Non linearity**	**Stride**
224^2^ × 3	Conv2d, 3 × 3	-	16	-	H-Swish	2
112^2^ × 16	Bneck, 3 × 3	16	16	√	ReLU	2
56^2^ × 16	Bneck, 3 × 3	72	24	-	ReLU	2
28^2^ × 24	Bneck, 3 × 3	88	24	-	ReLU	1
28^2^ × 24	Bneck, 5 × 5	96	40	√	H-Swish	2
14^2^ × 40	Bneck, 5 × 5	240	40	√	H-Swish	1
14^2^ × 40	Bneck, 5 × 5	240	40	√	H-Swish	1
14^2^ × 40	Bneck, 5 × 5	120	48	√	H-Swish	1
14^2^ × 48	Bneck, 5 × 5	144	48	√	H-Swish	1
14^2^ × 48	Bneck, 5 × 5	288	96	√	H-Swish	2
7^2^ × 96	Bneck, 5 × 5	576	96	√	H-Swish	1
7^2^ × 96	Bneck, 5 × 5	576	96	√	H-Swish	1
7^2^ × 96	Conv2d, 1 × 1	-	576	√	H-Swish	1
7^2^ × 576	Pool, 7 × 7	-	-	-	-	1
1^2^ × 576	Conv2d, 1 × 1, NBN	-	1280	-	H-Swish	1
1^2^ × 1280	Conv2d, 1 × 1, NBN	-	k	-	-	1

NBN represents no batch normalization. “-” represents not available. Bneck represents bottleneck. “Exp size” represents the size of the expanded dimension. “Out” represents the output feature matrix channel. “SE” represents squeeze-and-excitation.

### Mask output based on the optimized mask RCNN

The task of instance segmentation is different from object detection. The former not only needs to find all target fruits in the image, but also needs to segment each target fruit accurately. Based on the optimized mask RCNN, a two-stage segmentation model can accurately locate all object instances in the image and provide high-quality mask segmentation for each instance. The features extracted from the input image through MobileNetv3 and FPN structure are used for subsequent classification branch, regression branch, and mask branch. The implementation process can be summarized as the following three steps: (1) The extracted feature map generates region proposals in the feature map through RPN and performs candidate box regression and category differentiation for each region proposal. (2) The ROI align operation was used to align the features of the extracted ROI, the ROI to a fixed size was cut, and the feature map got after all ROI regularization. (3) The obtained feature map is subjected to boundary box regression and category prediction through the Fc layer. At the same time, additional mask branch is added to realize semantic segmentation of ROI and generate binary mask.

**Figure 5:**
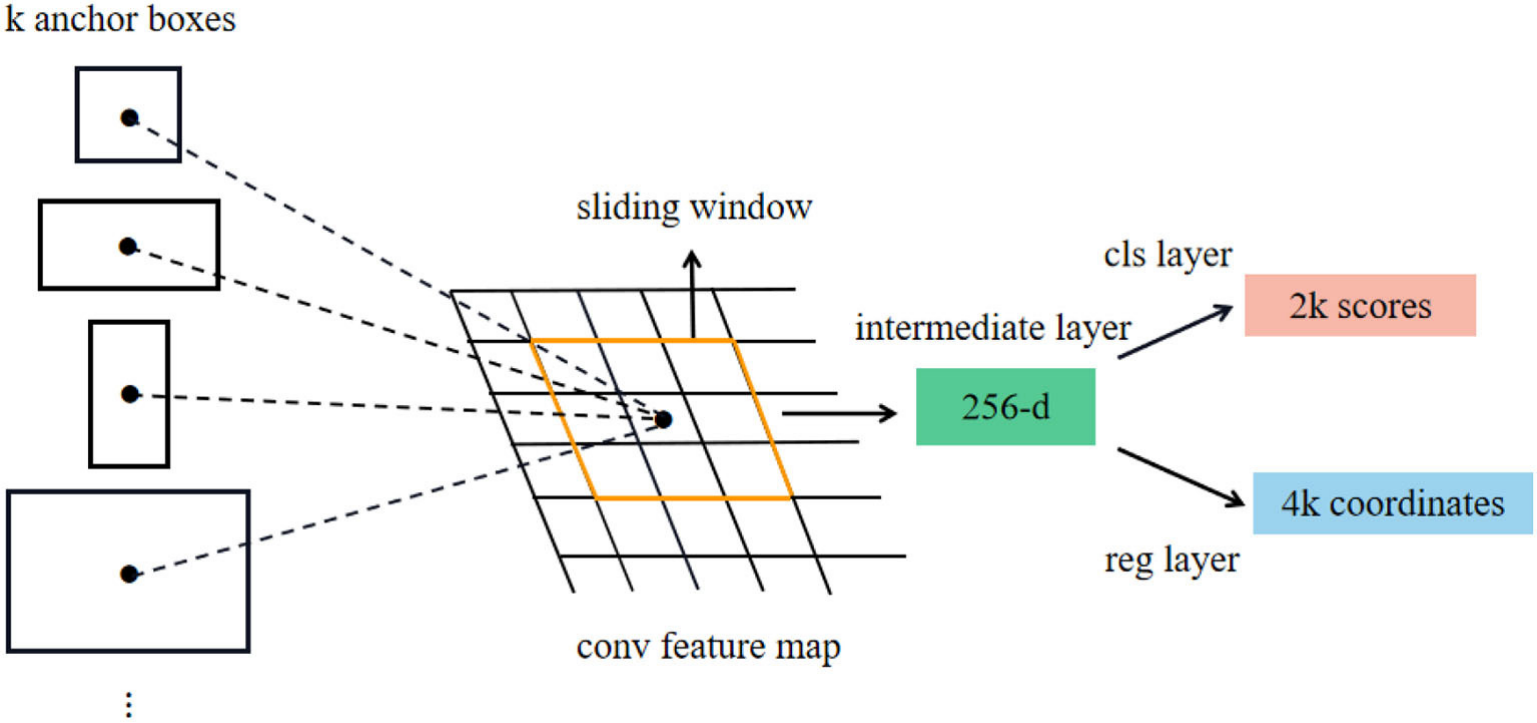
Structure diagram of RPN.

As shown in Figure 2, step 1 takes the features extracted from the backbone as the input of the region proposal network RPN and generates a region proposal. The specific process is shown in Figure 5. The feature map extracted from the backbone is used as the input of RPN, and *k* candidate windows (*k* = 9, 9 windows have 3 different shapes and 3 different aspect ratios) are set on each vector of the input feature map using a sliding window of 3 × 3 size as the initial detection box, namely, anchors. The size of each feature map is *H* × *W* × 256; that is, each vector is 256 dimensions and has *k* anchors. Each anchor should be distinguished between positive and negative samples. A 256-dimensional vector passes through the classification layer to obtain 2*K* score (i.e., the confidence of anchor's foreground and background). Then, through the regression layer, we get (*x, y, w*, and *h*) 4K offset coordinates regressed with the ground truth. RPN structure uses softmax (Horiguchi et al., 2019) layer as classifier to judge whether anchors belong to positive samples or negative samples and then uses boundary box regression to modify the anchor box to obtain accurate proposals.

The region proposals generated by RPN and the features extracted from the backbone are used as the input of ROI align layer in step 2. After synthesizing this information, proposal feature maps are extracted. Unlike the ROI pooling operation in faster RCNN (Bai et al., 2020), mask RCNN uses ROI align layer and bilinear interpolation algorithm to output the coordinates of pixels, which can avoid the problem of position quantization error caused by ROI pooling. Because ROI pooling extracts a 7 × 7 size feature map from each ROI, it is necessary to quantify the ROI of floating-point numbers into cells, which will lead to the error between ROI and extracted features and reduce the accuracy of the predicted mask. The application of ROI align greatly improves the quality of mask segmentation and improves the recognition accuracy of target fruit based on optimized mask RCNN. The fixed size ROI obtained by ROI align operation is used as the input of the Fc layer, and each ROI is subjected to category prediction and detection box regression. The specific process is shown in Figure 2. The extracted ROI obtains the confidence of the category and the offset of the coordinates, respectively, through the classification branch and the regression branch. At the same time, the model adds mask branch to divide ROI into binary mask; that is, ROI realizes semantic segmentation through fully convolutional network to obtain rough instance mask.

The new model realizes the goal of locating and segmenting the target fruit on the image through three branches: classification branch, regression branch, and mask branch. At this time, the optimized mask RCNN has obtained the preliminary segmentation mask, but the quality of binary segmentation mask still has a lot of room to improve.

### Boundary patch refinement post-processing module

The traditional mask RCNN has low feature spatial resolution and a small proportion of boundary pixels. These problems lead to the segmentation result of the target fruit that is not fine enough, resulting in rough boundary shape. The new model uses BPR as the post-processing module. BPR uses the strategy of cropping first and then refinement to optimize the rough boundary and correct the wrong pixels at the boundary of the target fruit. Due to the unique performance of instance contour mask optimization, BPR module can effectively improve the recognition rate of target fruits with serious occlusion and overlap and reduce the occurrence of false detection and missed detection. The refinement module first extracts dense small boundary patches for the instance boundary predicted by the optimized mask RCNN and then optimizes the boundary image patches through the refinement network with higher resolution.

**Figure 6:**
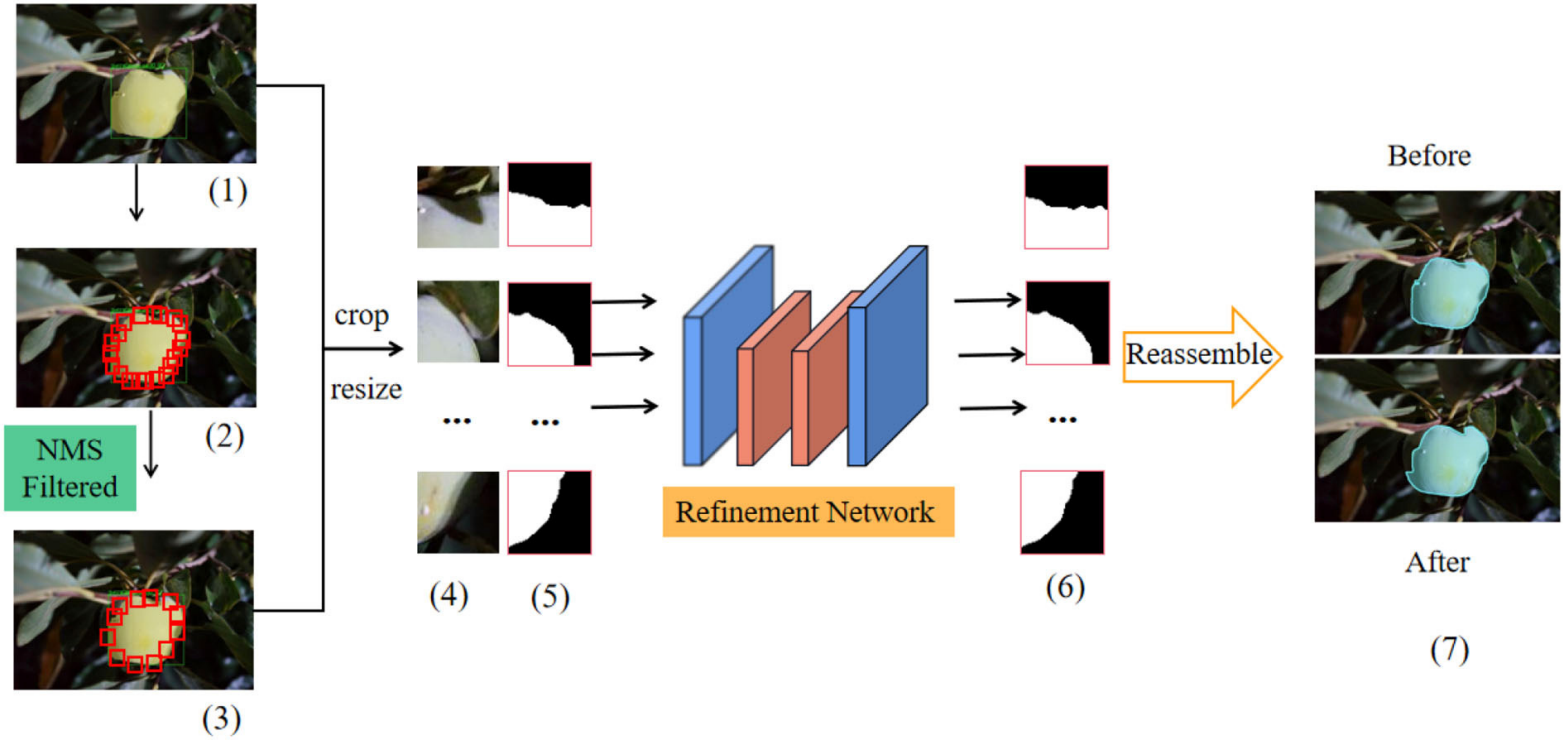
Overall structure diagram of BPR. Where (5) and (6) represent rough mask patches and refined mask patches respectively. “After” represents the result graph after mask optimization. “Before” represents the result before the rough mask optimization.

As shown in Figure 6, first, the initial rough segmentation result [shown in Figure 6 (1)] generated based on the optimized mask RCNN model is used as the input of the BPR module. Then, a large number of boundary patches are densely distributed along the boundary of the target fruit using the sliding window algorithm [shown in the red rectangle in Figure 6 (2)]. The central area of the rectangular boundary patch covers the pixels of the instance boundary, but these boundary patches contain a lot of redundant information, resulting in a lot of unnecessary calculations. Therefore, the non-maximum suppression (Karthikeyan et al., 2021) (NMS) algorithm (the threshold of NMS is set to 0.25) is used to filter out some redundant and overlapping boundary patches [shown in Figure 6 (3)], so as to take into account the accuracy and speed of the algorithm. After NMS algorithm, the corresponding mask patches were extracted [shown in Figure 6 (5)] from the reserved image patches [shown in Figure 6 (4)], these patches were cut into the same size, and they were used as the input of the refinement network at the same time. The mask patch extracted from the rough instance mask edge provides the location and semantic information for the refinement network and avoids the repeated learning of the refinement network. In this way, the refinement network only needs to learn how to locate the hard pixels around the decision boundary and locate them in the correct position.

The BPR module uses high-resolution network v2 (Sun et al., 2019) (HRNetV2) as the refinement network for binary semantic segmentation of each extracted boundary patch. HRNetV2 realizes the feature fusion of high and low resolutions. After sampling the low-resolution feature map, it is spliced with the high-resolution feature map. After convolution, the softmax layer generates the segmentation prediction map. As a refinement network, HRNetV2 makes the processing of boundary patches that have high resolution, so as to improve the quality of mask. Note that in this model, the number of input channels is 4 (RGB image block is 3 + mask block is 1), and the number of output channels is 2 (background color and foreground color). Moreover, the extracted mask patches provide positioning and semantic information for the refinement network. The refinement network only needs to correctly classify the wrong pixels at the boundary extracted by NMS algorithm, which speeds up the training convergence. All the refined mask patches (as shown in Figure 6 (6)] are reassembled into the instance boundary to obtain high-resolution expression and high-quality instance segmentation results. Figure 6 (7) shows the comparison of the optimized segmentation results with the pre-assembly. In the BPR training process, the model only extracts boundary patches from the target instances with IOU > 0.5 of prediction mask and real target mask (other target instances still participate in the inference process). The corresponding real mask patch uses pixel-level binary cross-entropy loss to supervise the output of the refinement network.

Overlapping target fruits inevitably share the same boundary patch, but the learning objectives are different. This problem can be solved by combining different mask patches for each instance. When the two target fruits overlap, the boundary patches overlap. The final result is to take the average value and use 0.5 as the threshold to judge whether a pixel belongs to the foreground or background. BPR model can correct the wrong segmentation of the boundary pixels of the target instance, and to a certain extent, it can distinguish the target fruits with overlapping and branches and leaves, so as to avoid the wrong scene of identifying two overlapping fruits as one fruit. The refinement network improves the accuracy and stability of the model to identify the target fruit and obtains high-quality target fruit segmentation results.

### Loss function

Loss function is the most basic and key element in deep learning. It can measure the quality of model prediction, show the gap between predicted value and actual data value, and make the model obtain optimal and faster convergence. Choosing the correct loss function can help the model learn how to focus on the correct set of features in the data. The optimization-based mask RCNN uses the sum of the loss functions of multiple tasks as the final loss function. The loss function consists of L_cls_, L_reg_, L_mask_ three parts, using Log loss, Smooth L1 loss, and binary cross-entropy loss, respectively, and the overall loss function is defined as Equation (2):


$$\begin{array}{l} L(\{p_{i}\},\{t_{i}\},\{\omega \})\;=\;\frac{1}{N_{cls}}\Sigma_{i}(-log[p_{i}^{*}p_{i}+(1-p_{i}^{*})(1-p_{i})])\\ \;+\;\lambda \frac{1}{N_{reg}}\Sigma_{i}p_{i}^{*}R(t_{i}-t_{i}^{*})-\;\\ \;\Sigma_{i}[p_{i}^{*}\;*ln(\sigma_{i})+(1-p_{i}^{*})\;*ln(1-\sigma_{i})] \end{array}$$

where p_i_ represents the probability of anchor i being predicted as an object, t_i_ represents the vector of bounding box coordinates for predicted anchor i, and ω represents the set of weights solved. $p_{i}^{*}$ stands for ground truth object label and $t_{i}^{*}$ stands for true box coordinate. N_cls_ represents the number of anchors in minibatch. N_reg_ represents the number of anchor locations. R represents smooth L1 loss function. σ_*i*_ is the return value of the sigmoid function calculated on sample i, based on the parameter ω. Regarding the definition of R and $p_{i}^{*}$ in Equation (2) as shown in Equation (3) and Equation (4), respectively.


(2) $$p_{i}^{*}=\{\begin{array}{c} 0,\;negative\;label\\ 1,\;positive\;label \end{array}$$

(3) $$R=Smooth_{L1}(x)=\;\{{}_{|x|-0.5,\;otherwise}^{\;0.5x^{2},\;if\;|x|<1}$$

where $x=t_{i}-t_{i}^{*}$.

## Data analysis and model comparison

### Experiment operation platform

The experiment was performed on a personal computer, with a processor of Inter (R) Core (TM) i5-7200U, and Radeon™ R7 M445 graphics card with an 8GB of memory. The software environment was Ubuntu 16.04, python 3.6, and pytorch 3.7. The hardware environment is Intel i7-8700K, random access memory 16G, and Nvidia GeForce GTX 1080 Ti Graphics Processing Unit (GPU).

### Model training

**Figure 7:**
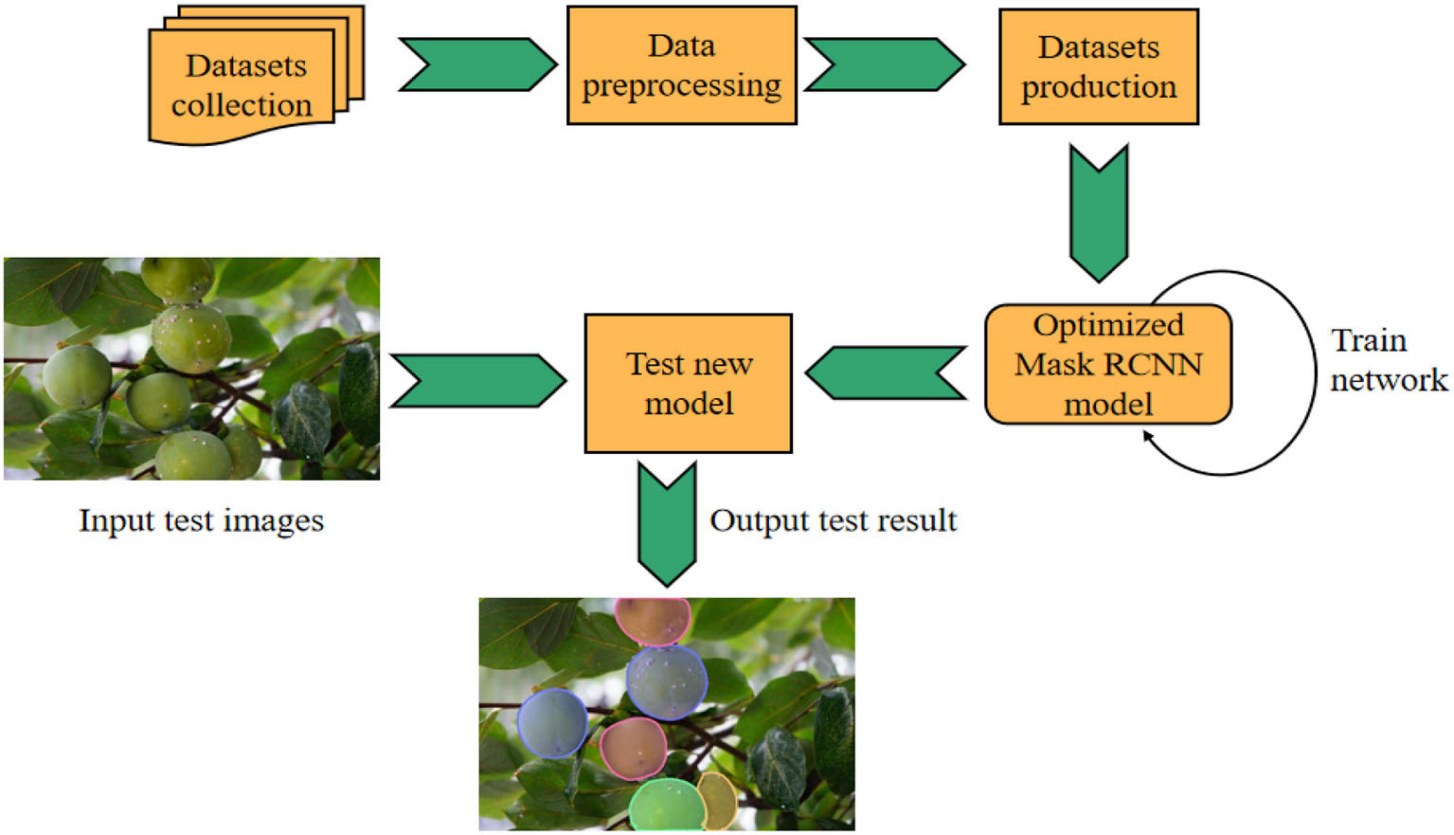
Overall experimental flow chart based on optimized mask RCNN model.

The whole process of experimental research includes data collection, dataset production, model training, and testing. The specific flow chart is shown in Figure 7. In the process of model training, the setting of super parameters plays an important role in model training and optimization.

The new model adopted initial weight of preliminary training based on common objects in context datasets, which helps to stabilize the loss function and improve the training accuracy. For model training, 10 epochs were iteratively trained using minibatch and the total number of iterations was 16,000. In addition, the initial learning rate was set to 0.0025, the decay to 0.0001, and the momentum to 0.9. The learning rate will control the learning progress of the model in the iterative process. In the gradient descent method, the given unified learning rate is given, and the whole optimization process is updated with the determined step size. In the early stage of iterative optimization, if the learning rate is large, the forward step size will be longer. At this time, the gradient descent can be carried out at a faster speed, and in the later stage of iterative optimization, the value of learning rate and step size will be gradually reduced, This will help the convergence of the algorithm and make it easier to approach the optimal solution. The number of iterations refers to the number of times, the whole training set is inputted into the network for training. When the difference between the test error rate and the training error rate is small, the current number of iterations can be considered appropriate.

### Evaluation index

Selecting the appropriate evaluation method in the research process can quickly find the possible problems in the process of model selection and training and iteratively optimize the model. In this study, the average precision (AP) and average recall (AR) are used to evaluate the performance of the instance segmentation model. Precision refers to the proportion of the number of positive samples correctly classified by the classifier to the number of positive samples classified by the classifier. Recall refers to the proportion of the number of positive samples correctly classified by the classifier to the number of all positive samples. The calculation method of accuracy rate and recall rate is shown in formula (5) and formula (6):


(4) $$\begin{array}{rc} Precision & =\frac{TP}{TP+FP} \end{array}$$

(5) $$\begin{array}{rc} Recall & =\frac{TP}{TP+FN} \end{array}$$

where TP represents the positive sample predicted as positive by the model, FP represents the negative sample predicted as positive by the model, and FN represents the positive sample predicted as negative by the model.

Under different IOU thresholds, the values of precision and recall will also change. Average precision summarizes the shape of the accuracy/recall curve and is defined as the average of the accuracy over a set of 11 equally spaced recall levels [0, 0.1, 0.2,..., 1]. The average accuracy can be expressed as formula (7):


(6) $$\begin{array}{r} AP=\overset{1}{\underset{0}{\int }}p\left(r\right)dr \end{array}$$

where *p* (*r*) is a function with r as a parameter.

In fact, this integral is very close to the change of precision value multiplied by recall value for each threshold and then accumulate the products obtained under all thresholds. As shown in formula (8):


(7) $$\begin{array}{r} AP\approx \Sigma_{k=1}^{N}P\left(k\right)\Delta r\left(k\right) \end{array}$$

where *N* represents the number of all pictures in the test set, P (k) represents the value of precision when *k* pictures can be recognized, and Δ*r*(*k*) represents the change of recall value when the number of recognized pictures changes from *k* – 1 to *k*.

### Result and analysis

#### Segmentation effect of green target fruit

**Figure 8:**
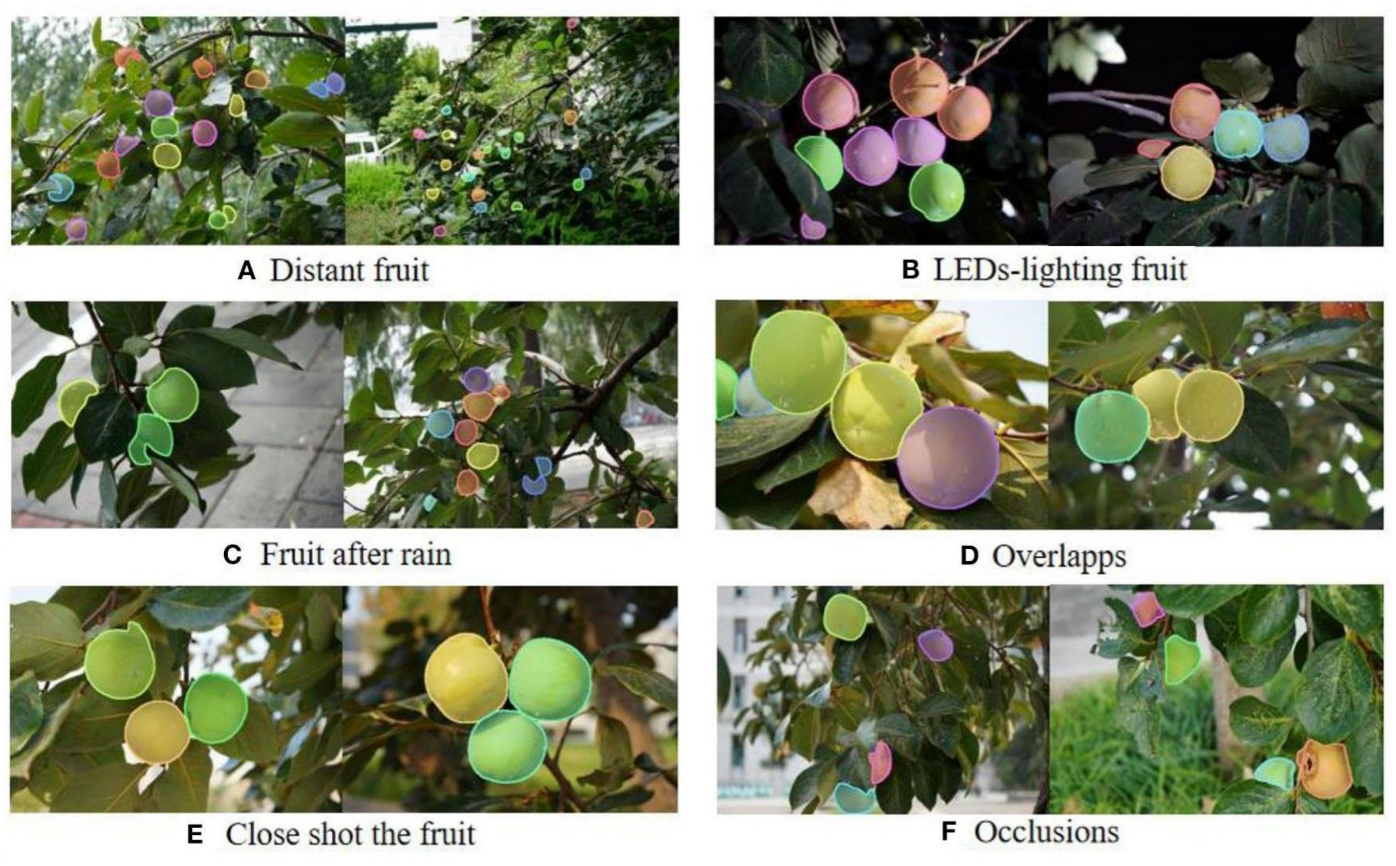
Visual diagram based on optimized mask RCNN test. **(A)** Distant fruit, **(B)** LEDs-lighting fruit, **(C)** Fruit after rain, **(D)** Overlapps, **(E)** Close shot the fruit, **(F)** Occlusions.

After the network training based on the optimized mask RCNN model is completed, the optimal model is selected based on mAP index, and the performance of the new model is comprehensively evaluated. The average precision and average recall of the new model based on persimmon dataset are recorded in Table 3. Figure 8 shows the visual segmentation results of some datasets, including the segmentation results of green persimmons in different complex orchard environments such as night, rainy day, occlusion, overlap, and distant view. This paper especially selects the visual pictures with complex environment as the representative, and each complex condition is displayed with two different pictures. Note that each condition marked in the figure may contain a variety of complex situations, such as distant view, occlusion, and overlap in Figure 7A below.

**Table 3:** Based on the optimized mask RCNN model, the average precision, and average recall of persimmon datasets at different thresholds, sizes, and quantities are analyzed.

**Metric**	**mAP**	**AP_50_**	**mAP_S_**	**mAP_M_**	**mAP_L_**	**mAR**	**mAR_S_**	**mAR_M_**	**mAR_L_**
Value (%)	76.3	93.0	33.2	78.4	90.7	81.1	45.0	83.3	92.5

The lower right corner of the evaluation character in the table represents the size of the target fruit. S represents the small size of the target fruit; that is, the range of the rectangular area is [0, 32^*^32]. M represents medium size target fruit; that is, the range of rectangular area is [32^*^32, 96^*^96]. L represents the large size target fruit; that is, the range of the rectangular area is [96^*^96, ∞]. The number in the lower right corner of the character indicates that the threshold of IoU is 0.5.

It can be seen from the Figure 8 that the target fruit often appears in complex and difficult scenes, just as a target fruit will appear many unstable factors such as occlusion, unclear light, rainy days, color system with background leaves, and so on. The complex and changeable orchard environment brings great challenges to fruit and vegetable picking. However, from the segmentation results, the new model has less missed detection and false detection for the target fruit in various complex scenes, and the positioning of the target fruit is accurate, which meets the needs of high-quality segmentation. Even if the target fruit is not annotation due to shooting blur and other reasons, it can still be segmented. Therefore, the optimized mask RCNN model overcomes the difficulties caused by incomplete boundary information and the similarity between fruit color and leaf color and has strong generalization ability and robustness.

Table 3 shows the different average precision and average recalls obtained from the green persimmon dataset under different IOU thresholds and quantities and target fruit size. According to the analysis in Table 3 and Figure 8, the new model has the best effect on large target fruit segmentation (AP achieved 90.7%), and all targets in the picture can be recognized and achieve high-quality segmentation. For the fruit with small vision, the segmentation effect is slightly worse (AP value is 33.2%), but most of the fruit can still be completely recognized, and only the objects at the boundary of the image or blurred cannot be recognized. According to Figure 8, it can be observed that when the target fruit is covered and overlapped by branches and leaves (as shown in Figures 8F,D), although only a small part of the target is exposed, it can still be accurately identified. Combined with the analysis of tables and images, it can be concluded that: The distribution of prospective target fruits is dense, the target size is small, and it is easy to have large area occlusion and overlap, resulting in the serious lack of boundary information. This is the biggest difficulty in intelligent picking. However, in terms of the overall segmentation effect, the optimized mask RCNN model is ideal for the segmentation of target fruits in various complex scenes. Even for the heavily occluded and overlapping target fruits, it still achieves high-precision and high-quality segmentation mask. Thanks to the powerful backbone network and unique post-processing module of the new model, it can better process edge information and improve resolution. Even for fruits with incomplete edge information, it can still achieve accurate positioning and high-quality segmentation.

#### Algorithm comparison

The new model takes mask RCNN as the baseline, carries out a series of optimization, and achieves high accuracy and high-quality segmentation mask. Moreover, in the process of experimental design, this study fully considered the objectivity and authenticity of experimental evaluation. Therefore, ablation experiment was adopted to fully verify that the new model has higher accuracy and higher quality for green target fruit. In this study, the new model is compared with four algorithms: baseline mask RCNN, SOLOv2 (Wang et al., 2020), YOLACT (Bolya et al., 2019), and Cascade_RCNN (Cai and Vasconcelos, 2019). Among them, mask RCNN model and Cascade_RCNN model are the two-stage instance segmentation methods, whereas SOLOv2 and YOLACT are one-stage segmentation models. To ensure the objectivity and effectiveness of the comparative experiment, the same super parameters will be set in the experimental process, and the same persimmon dataset will be used for testing. The data results of the test are entered as shown in Table 4.

**Table 4:** Comparison of instance segmentation performance of five different networks.

**Metric (%)**	**mAP**	**AP_50_**	**mAP_S_**	**mAP_M_**	**mAP_L_**	**mAR**	**mAR_S_**	**mAR_M_**	**mAR_L_**
SOLOv2	58.9	85.1	18.7	63.2	68.7	67.9	29.9	70.7	78.2
Mask RCNN	73.2	90.6	29.6	75.2	87.6	77.4	41.6	79.3	90.3
YOLACT	64.6	88.6	20.0	67.1	81.2	72.5	34.5	74.2	86.9
Cascade_RCNN	71.4	90.7	23.9	72.7	88.5	75.5	36.9	77.2	90.6
Ours	76.3	93.0	33.2	78.4	90.7	81.1	45.0	83.3	92.5

As shown in Table 4, the optimized mask RCNN leads other models with average recall of 81.1% and average precision of 76.3%, respectively. Compared with the baseline mask RCNN, the AP and AR values of the new model increased greatly. Moreover, the recognition rate of small target fruit in the new model is significantly higher than that in other models, especially for the recall rate. Through further analysis of the experimental results, it is found that due to the efficient post-processing operation of BPR module, the phenomenon of repeatedly identifying overlapping multiple targets as a whole and identifying leaves as fruits is reduced in the new model. This shows that the new model reduces the false detection rate and missed detection rate to a certain extent. The experimental data show that a series of optimization of mask RCNN greatly improves the performance of the model, which fully proves the feasibility of this research method. The dataset of this study contains a large number of complex scenes, but the model still achieves satisfactory average precision and average recall, which can meet the requirements of application to the field of agricultural intelligent picking.

**Figure 9:**
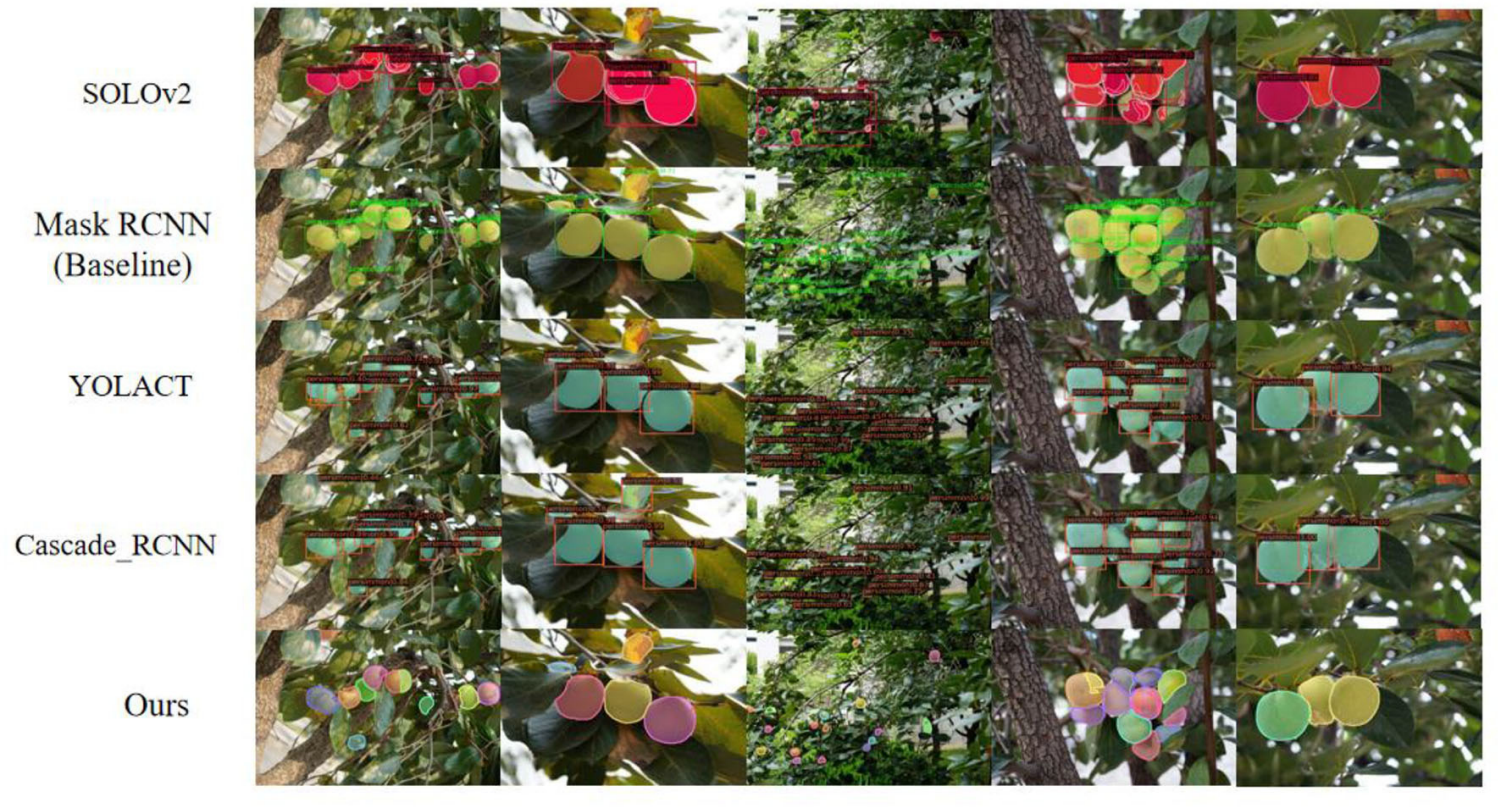
Comparison of segmentation effects of five different models.

As shown in Figure 9, the segmentation effects of five different models are shown. According to the analysis of Figure 9 and Table 4, SOLOv2 has the worst effect, the segmentation effect of the experiment is not clear, the boundary shape is incomplete, and there are a lot of missed detection. The experimental results based on the optimized mask RCNN model are the best. Both the accuracy and the quality of segmentation are better than other models. The segmentation quality of YOLACT and Cascade_RCNN models is relatively good, but they are prone to missed detection and false detection, and the accuracy is far lower than that of the new model. The recognition accuracy of mask RCNN model is second only to the new model, but the segmentation quality of mask RCNN model is poor and the processing of edge pixels is not accurate enough. It is very easy to repeatedly recognize multiple target fruits as a whole. This paper has important research value for the optimization of mask RCNN. At the same time, the model is optimized from the two aspects of segmentation quality and recognition accuracy, so as to realize the real high-quality accurate segmentation. Based on the performance of optimized mask RCNN model with high accuracy, strong robustness, good generalization ability, and high segmentation quality, it is of great significance to deploy to the field of intelligent agriculture.

#### Ablation study

To verify the effectiveness of BPR module, this study conducted additional ablation experiments based on the persimmon dataset. In the training, the NMS threshold of BPR modules added to all models is set to 0.25. In the inference, set NMS to 0.55. HRNetV2_W18_Small is used as the optimized network of BPR module, and the size of the extracted boundary patches is 64 ^*^ 64 without padding. Then, boundary patches are resized to 256 ^*^ 256 as the input of BPR. In this experiment, the influence of BPR module on the accuracy and recall of the model is verified by controlling the variable BPR module while keeping other settings unchanged.

This study used three different models to verify the effectiveness of BPR module, including one-stage and two-stage segmentation models. The experimental results are shown in Table 5. By analyzing the data in Table 5, it was found that the model with the addition of the BPR module had higher AP and AR, which could improve the recognition of target fruits at different scales. Moreover, BPR module can be easily integrated into most pixel-based instance segmentation models and greatly improve the segmentation accuracy of the model. Thus, BPR, as a plug and play module, can easily help most models improve accuracy.

**Table 5:** The effect comparison of three different models with or without BPR module.

**Metric (%)**	**mAP**	**AP_50_**	**mAP_S_**	**mAP_M_**	**mAP_L_**	**mAR**	**mAR_S_**	**mAR_M_**	**mAR_L_**
SOLO	37.8	60	5.3	36.6	62.1	49.4	10.4	49.2	72.1
SOLO^+^	39.7	62.3	7.1	39	64.5	53	12.7	51.2	74.6
D2Det	58	83.2	40.6	65	87.8	61.9	45.9	69.1	89.6
D2Det^+^	61.1	86	43.3	67.9	90.1	65.1	48.8	72	92.7
Mask RCNN	73.2	90.6	29.6	75.2	87.6	77.4	41.6	79.3	90.3
Mask RCNN^+^	75.8	92.9	32.5	77.9	89	80.6	44.5	82	92.1

“+” represents that BPR module is added to the model.

## Conclusion

Based on the classic mask RCNN algorithm, this paper optimizes and innovates the new model to achieve ideal recognition accuracy and segmentation quality in complex orchard environment. The new model applied MobileNetv3 as a lightweight backbone network, which is able to reduce the number of parameters in the mask RCNN model and achieve a significant improvement in the accuracy of identifying target fruits without sacrificing speed! MobileNetv3, as a lightweight backbone network, can further meet the storage resource requirements of automated picking robots and can be easily applied to similar inspection or segmentation networks. The features extracted by the backbone network pass through the subsequent classification, regression, and mask segmentation branches to obtain the coarse segmentation mask. The boundary patches of the rough segmentation mask are used as the input of the post-processing module BPR. The extracted boundary patches are optimized by crop-then-refine strategy, which significantly improves the resolution and segmentation quality. Although the new model has achieved satisfactory results, there are still some rooms for progress:

(1) The experimental dataset used in the new model contains fewer pictures, so we should consider expanding the dataset in the future.

(2) The new model as a two-stage anchor segmentation model, the size of the anchor limits the development of the model, and the association between the presence and absence of the anchor frame should be further searched.

## Data availability statement

The original contributions presented in the study are included in the article/supplementary material, further inquiries can be directed to the corresponding author/s.

## Author contributions

WJ: conceptualization, writing—original draft preparation, and funding acquisition. JW: data curation, software, and writing—original draft preparation. QZ: data curation and visualization. NP: data curation and software. YN: methodology, software, and validation. XY: conceptualization and writing—reviewing and editing. YD: validation and visualization. XG: conceptualization, funding acquisition, and writing—reviewing and editing.

## Funding

This work was supported by the Natural Science Foundation of Shandong Province in China (No.: ZR2020MF076), the National Nature Science Foundation of China (No.: 81801776), the Natural Science Foundation of Jiangsu Province (No.: BK20170256), the Focus on Research and Development Plan in Shandong Province (No.: 2019GNC106115), the Opening Foundation of Key Laboratory of intelligent control and manufacturing of agricultural machinery (Wuyi University) Fujian Province University (AMICM202104).

## Conflict of interest

The authors declare that the research was conducted in the absence of any commercial or financial relationships that could be construed as a potential conflict of interest.

## Publisher's note

All claims expressed in this article are solely those of the authors and do not necessarily represent those of their affiliated organizations, or those of the publisher, the editors and the reviewers. Any product that may be evaluated in this article, or claim that may be made by its manufacturer, is not guaranteed or endorsed by the publisher.
